# Genome-wide characterization and expression profiling of MAPK cascade genes in *Salvia miltiorrhiza* reveals the function of SmMAPK3 and SmMAPK1 in secondary metabolism

**DOI:** 10.1186/s12864-020-07023-w

**Published:** 2020-09-14

**Authors:** Yongfeng Xie, Meiling Ding, Bin Zhang, Jie Yang, Tianlin Pei, Pengda Ma, Juane Dong

**Affiliations:** 1 College of Life Sciences, Northwest A&F University, Yangling, China; 2 Shanghai Key Laboratory of Plant Functional Genomics and Resources, Shanghai Chenshan Botanical Garden, Shanghai Chenshan Plant Science Research Center, Chinese Academy of Sciences, Shanghai, China

**Keywords:** *Salvia miltiorrhiza*, Gene family, MAPK cascades, Co-expression analysis, Phenolic acid synthesis, Tanshinone synthesis

## Abstract

**Background:**

The contribution of mitogen-activated protein kinase (MAPK) cascades to plant growth and development has been widely studied, but this knowledge has not yet been extended to the medicinal plant *Salvia miltiorrhiza,* which produces a number of pharmacologically active secondary metabolites.

**Results:**

In this study, we performed a genome-wide survey and identified six MAPKKK kinases (*MAPKKKKs*), 83 MAPKK kinases (*MAPKKKs*), nine MAPK kinases (*MAPKKs*) and 18 MAPKs in the *S. miltiorrhiza* genome. Within each class of genes, a small number of subfamilies were recognized. A transcriptional analysis revealed differences in the genes’ behaviour with respect to both their site of transcription and their inducibility by elicitors and phytohormones. Two genes were identified as strong candidates for playing roles in phytohormone signalling. A gene-to-metabolite network was constructed based on correlation analysis, highlighting the likely involvement of two of the cascades in the synthesis of two key groups of pharmacologically active secondary metabolites: phenolic acids and tanshinones.

**Conclusion:**

The data provide insight into the functional diversification and conservation of MAPK cascades in *S. miltiorrhiza*.

## Background

Plants have developed diverse strategies to protect themselves from pathogens and environmental stress, many of which are based on the production of secondary metabolites [1–4]. Metabolic engineering of natural product pathways is a feasible strategy over the years for enhancement of plant disease resistance [5]. Some of these compounds also have beneficial nutraceutical or pharmacological properties (classic bacteriostatic, antibiotic, antivirulence, anticancer, anti-diabetic, ect.) [6–9]. It has been estimated that at least 30% of therapeutic compounds in current use have been derived from medicinal plants [10]. The root of *S. miltiorrhiza* has a long history of use in Chinese herbal medicine as a source of compounds that are effective for curing a range of illnesses [2]. Its major bioactive compounds fall into two groups: hydrophilic phenolic acids and lipophilic tanshinones [9]. The former are synthesized in planta through both the phenylpropanoid and a tyrosine-derived pathways [11, 12], while the latter are generated through the cytoplasmic mevalonic acid pathway and the plastidial 2-C-methyl-D-erythritol-4-phosphate pathway [13]. Treating plants with the phytohormones have been shown to promote the accumulation of both phenolic acids [11] and tanshinones [13].

Mitogen-activated protein kinase (MAPK) cascades are a universal characteristic of eukaryotic cells. These cascades involve the activity of four distinct types of kinases: MAP kinases (*MAPKs*), MAPK kinases (*MAPKKs*), MAPKK kinases (*MAPKKKs*) and MAPKKK kinases (*MAPKKKKs*) [14–16]. MAPK cascades are important for plant growth [17], development [18–20] and defence against biotic [21, 22] and abiotic stress [23–25]. Furthermore, MAPK cascades are very likely to be involved in secondary metabolism, including camalexin [26], indole glucosinolate [26], nicotine [27], anthocyanin [28] and phytoalexin [29]. It has been reported that methyl jasmonate (MeJA), salicylic acid (SA), gibberellic acid (GA) and abscisic acid (ABA) regulate the accumulation of phenolic acids [11] and tanshinones [13] in *S. miltiorrhiza.* Furthermore, the MAPK cascade regulates the biosynthesis and signalling pathways of SA [22, 29, 30], JA [31–33], ABA [25, 31, 34–37], auxin (AUX) [17, 19] and ethylene (ETH) [17, 26]. The acquisition of an increasing number of whole plant genome sequences has revealed large numbers of genes encoding the component enzymes of MAPK cascades. For example, a total of 75 *MAPKKK* [38], 8 *MAPKK* [39], and 17 *MAPK* [40] genes have been reported in the rice genome, whereas the *Arabidopsis thaliana* genome contains 10 *MAPKKKK*, 80 *MAPKKK*, 10 *MAPKK* and 20 *MAPK* genes [15, 16]*.* It was also reported that 74 *MAPKKK*, 9 *MAPKK*, and 19 *MAPK* genes can be found in maize [41–43], whereas 89 putative *MAPKKK*, 6 *MAPKK*, and 16 *MAPK* genes are found in tomato [44, 45]. The present study takes advantage of the availability of a complete *S. miltiorrhiza* genome sequence [46] to document the species’ MAPK cascade gene content and was undertaken because these genes are likely important for the synthesis of its bioactive secondary metabolites. Their identification may lead to the application of metabolic engineering with a view to improving the productivity of the *S. miltiorrhiza* plant.

## Results

### The MAPK cascade enzymes encoded by *S. miltiorrhiza*

The HMMER-based search of the *S. miltiorrhiza* genome sequence [46] identified a total of six *SmMAPKKKKs*, 83 *SmMAPKKKs*, nine *SmMAPKKs* and 18 *SmMAPKs* using 10 *MAPKKKKs*, 80 *MAPKKKs*, 10 *MAPKKs* and 20 *MAPK* sequences from the *A. thaliana* genome as queries [15, 16]. The relevant sequences and gene ID are provided in Additional file 1 Table S1, while their key structural and other details are provided in Additional file 1 Table S2. The length of the predicted MAPKs ranged from 353 (SmMAPK7) to 690 (SmMAPK10) residues, that of the MAPKKs from 289 (SmMAPKK5) to 521 (SmMAPKK3) residues, that of the MAPKKKs from 185 (SmMAPKKK49) to 1401 (SmMAPKKK3) residues and that of the MAPKKKKs from 456 (SmMAPKKKK6) to 837 (SmMAPKKKK5) residues; the molecular weights in the full set of polypeptides ranged from 32.4 kDa (SmMAPKK5) to 155.3 kDa (SmMAPKKK3), and their pI ranged from 4.49 (SmMAPKKK38) to 9.77 (SmMAPKKK14).

### The phylogeny and exon-intron structure of the MAPK cascade genes

**Fig. 1:**
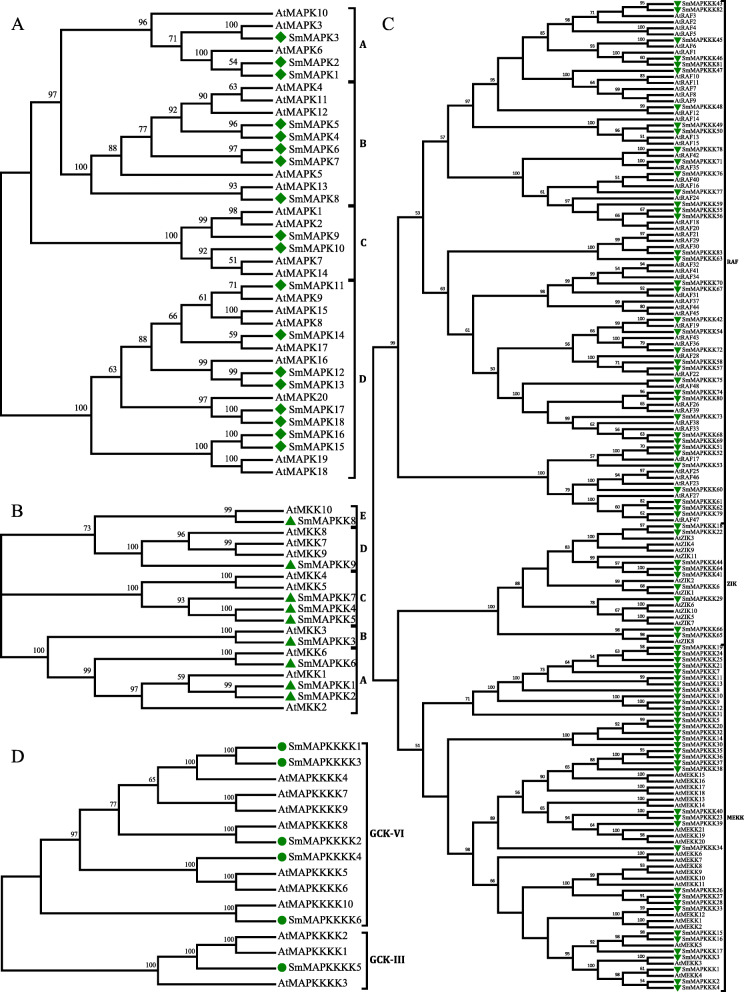
The phylogeny of the SmMAPK gene family. The dendrograms were constructed using the neighbour-joining method applied to full-length *A. thaliana* and S. miltiorrhiza MAPK sequences. Bootstrap (500 replicates) values appear at each branch. **a** MAPK sequences, **b** MAPKK sequences, **c** MAPKKKK sequences, **d** MAPKKK sequences

**Fig. 2:**
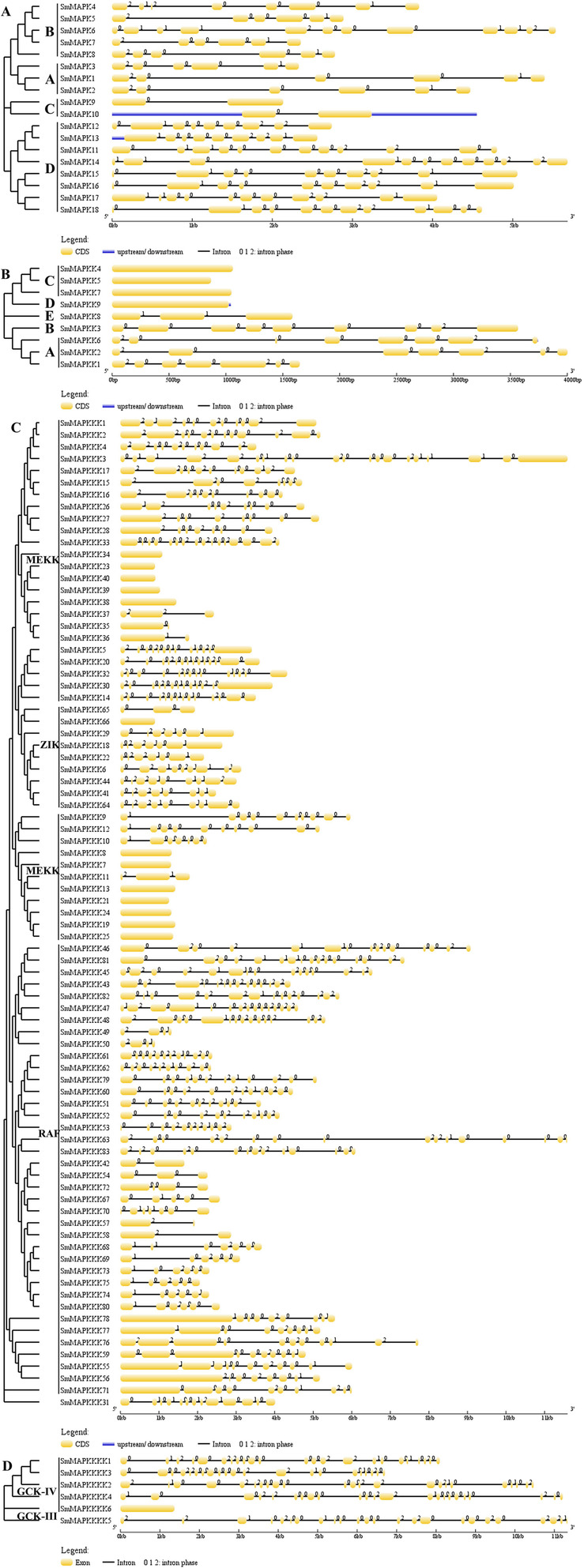
The intron/exon structure of the SmMAPK gene family members. **a** MAPK sequences, **b** MAPKK sequences, **c** MAPKKKK sequences, and **d** MAPKKK sequences. Exons are shown as yellow boxes and introns with a simple line. Untranslated regions are indicated by thick blue lines. 0, 1, and 2 represent the intron phase. Gene models are drawn to scale

The alignment of *S. miltiorrhiza* sequences with those of *A. thaliana* revealed that the *SmMAPKs* fell into four subfamilies (A through D), the *SmMAPKKs* into five subfamilies (A through E), the *SmMAPKKKs* into three subfamilies (MEKK, ZIK and RAF) and the *SmMAPKKKKs* into two subfamilies (GCK-III and -VI) (Fig. 1). The RAFs constituted the largest single subfamily (38 members), followed by the *MEKKs* (36 members); only a single *SmMAPKK* was present in each of subfamilies B, D and E, as was the case for subfamily D of the *SmMAPKs*. The variation in the exon-intron structure of the *S. miltiorrhiza* genes is illustrated in Fig. 2. The number of introns present among the *SmMAPKs* varied from one (*SmMAPK9*, *SmMAPK10*) to 11 (*SmMAPK6*, *SmMAPK11*, *SmMAPK14*, *SmMAPK18*); among the *SmMAPKKs* from zero (*SmMAPKK4*, *SmMAPKK5*, *SmMAPKK7* and *SmMAPKK9*) to eight (*SmMAPKK3*); among the *SmMAPKKKs* from zero (*SmMAPKKK7*, *SmMAPKKK8*, *SmMAPKKK13*, *SmMAPKKK19*, *SmMAPKKK21*, *SmMAPKKK23*, *SmMAPKKK24*, *SmMAPKKK25*, *SmMAPKKK34*, *SmMAPKKK38*, *SmMAPKKK39* and *SmMAPKKK40*) to 19 (*SmMAPKKK3*) and among the *SmMAPKKKKs* from zero (*SmMAPKKKK6*) to 23 (*SmMAPKKKK5*). The *SmMAPKs* in subfamily A all harboured six exons and showed similar gene lengths to one another, while the subfamily C members all harboured two exons.

### Motif content of SmMAPK enzymes

**Fig. 3:**
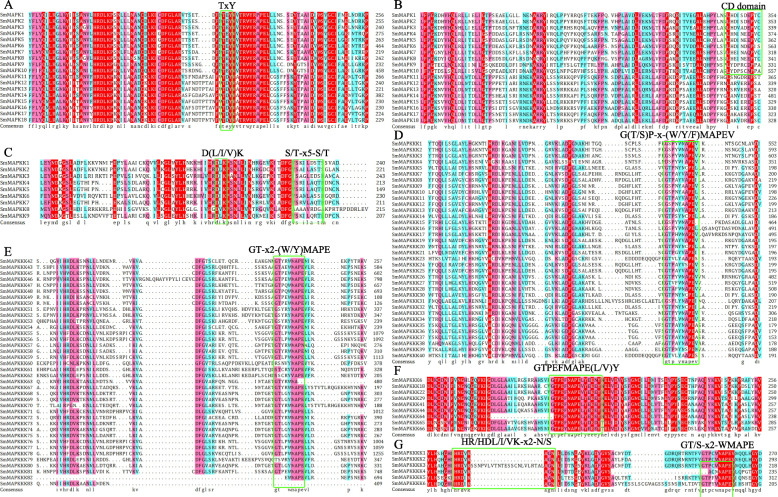
The domain content of the SmMAPK gene family products. **a** TxY, **b** CD domain, **c** D(L/I/V)K S/T-× 5-S/T, **d** G(T/S)P-x-(W/Y/F)MAPEV, **e** GT-× 2-(W/Y)MAPE, **f** GTPEFMAPE(L/V)Y, and **g** HR/HDL/I/VK-× 2-N/S GT/S-× 2-WMAPE

All of the predicted gene products included various characteristics associated with MAPK cascade enzymes. The SmMAPKs shared the conserved TxY motif (Fig. 3a) contained within the activation loop lying between subdomains VII and VIII as well as the (LH)DxxDE(P) x CD domain (Fig. 3b), which acts as the MAPKK docking site. The TxY motif in the members of subfamilies A, B and C was represented by TEY, and in subfamily D, it was represented by TDY. Each of the SmMAPKKs harboured a D(L/I/V) K motif along with the consensus sequence S/T-× 5-S/T (Fig. 3c). The latter motif was conserved across all members of subfamilies A through D, but the S/T site was altered to G/A in the subfamily E member SmMAPKK8. Among the MAPKKKs, the MEKKs all retained the conserved signature sequence G(T/S)P-x-(W/Y/F) MAPEV, the RAFs retained the GT-x-x-(W/Y) MAPE sequence, and the ZIKs retained the GTPEFMAPE(L/V) Y sequence (Fig. 3d-f). The two motifs shared by the SmMAPKKKKs were H-R/H-D-L/I/V-K-x-x-N/S (subdomain VIb) and G-T/S-x-x-W/Y/F-M/L/−A/S/P-P-E (subdomain VII) (Fig. 3g).

**Fig. 4:**
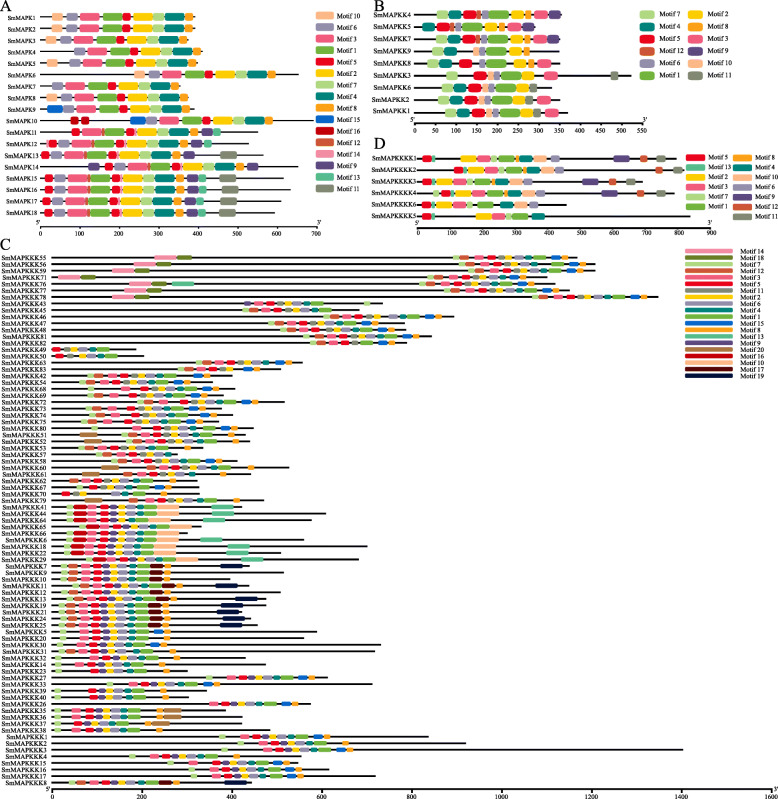
The motif content of the SmMAPK gene family products. **a**-**d** Putative motifs in **a**) MAPK, **b** MAPKK, **c** MAPKKKK, and **d** MAPKKK sequences, as predicted by MEME software

A MEME-based analysis of the full set of sequences confirmed the identity of each group. Thus, nine out of 16 motifs were common to and conserved in all of the SmMAPK group A proteins, 10 out of 12 in the SmMAPKK group C proteins, 11 out of 20 in the SmMAPKKK ZIKs and 9 out of 13 in all of the GCK-IV SmMAPKKKKs (Fig. 4 and Additional file 2 Figure S1). Seven motifs were conserved across all of the *S. miltiorrhiza* MAPKKKK proteins, including motifs #1 (G-T/S-x-x-W/Y/F-M/L/−A/S/P-P-E) and #3 (H-R/H-D-L/I/V-K-x-x-N/S). All of the GCK-IV MAPKKKK proteins except for SmMAPKKKK4 retained N-terminal motif #5, and all except for SmMAPKKKK6 retained C-terminal motif #11 (Fig. 4d and Additional file 2 Figure S1). Seven motifs (#1 through #5, #7 and #8) were reasonably well conserved among the SmMAPKs; motifs #9, #12 and #14 were only found in subfamily D members, and motif #15 was only found in subfamily C members (Fig. 4a). There were three conserved motifs (#1, #2 and #6) in the SmMAPKKs, but motif #9 was a subfamily C-specific motif (Fig. 4a). Motif #5 was reasonably well conserved among the SmMAPKKKs (Fig. 4c). There were seven conserved motifs (#1 through #5, #7 and #13) in the SmMAPKKKKs, but motifs #6 and #10 were only found in subfamily GCK-IV (Fig. 4d). The sequences 1500 nt upstream of the *S. miltiorrhiza* MAPK cascade genes harboured an array of cis acting elements (Additional file 1 Table S3).

### Coexpression of genes responsible for the synthesis of phenolic acids and tanshinones

**Fig. 5:**
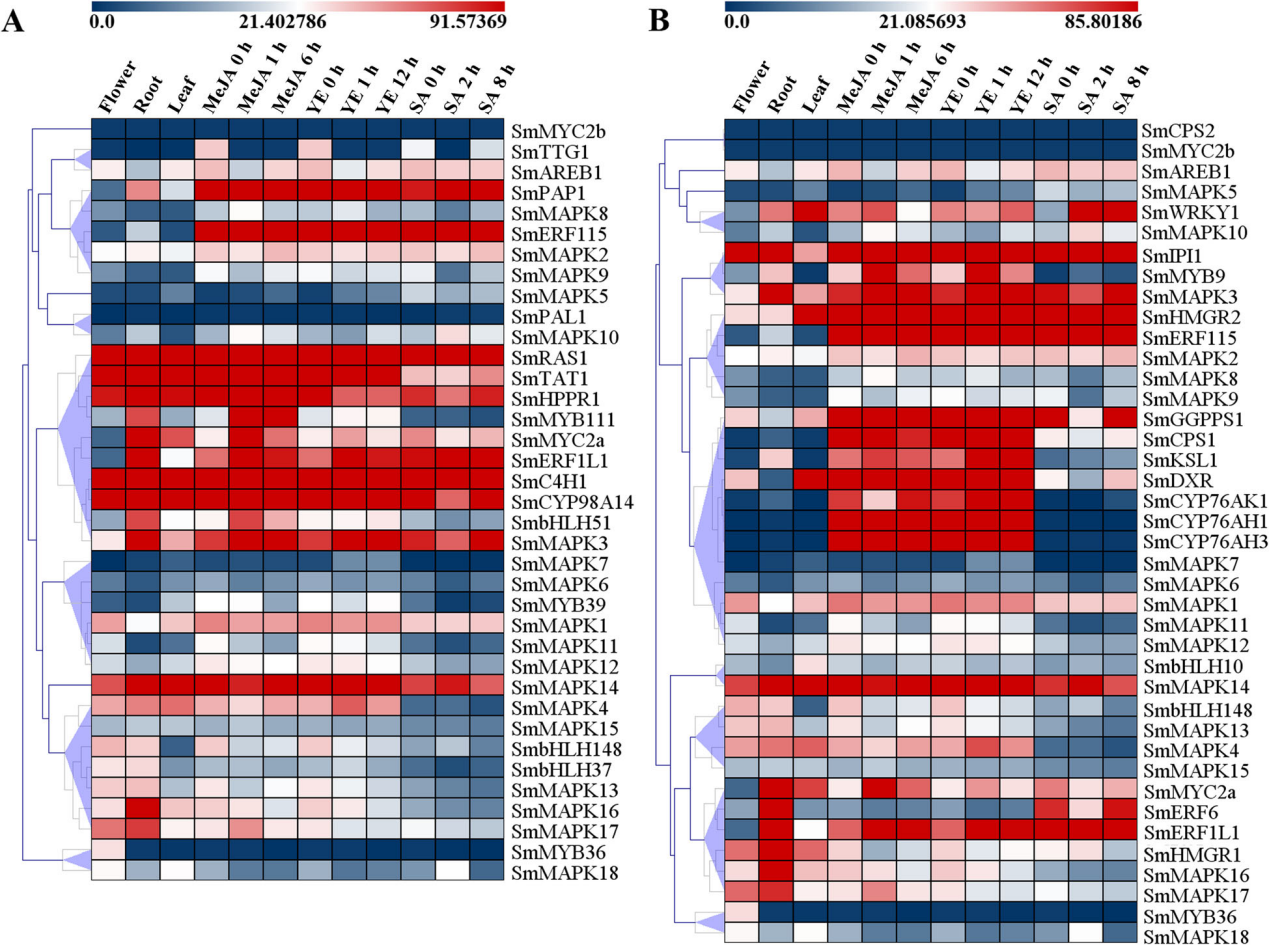
Heat maps illustrating patterns of gene coexpression. Genes encoding (**a**) key phenolic acid pathway enzymes, transcription factors and members of the SmMAPK family, **b** enzymes involved in the synthesis of tanshinones, transcription factors and members of the SmMAPK family. Transcript abundance was estimated in the roots, leaves and flowers of *S. miltiorrhiza* plants, some of which were exposed to salicylic acid, methyl jasmonate or yeast extract. Pearson correlation coefficient (PCC) values were calculated for these genes. Blue: low abundance, red: high abundance

The co-expression analysis directed toward genes (the information on enzymes and transcription factors is listed in Additional file 1 Table S4 and Table S5) encoding key phenolic acid pathway enzymes (*SmC4H1*, *SmCYP98A14*, *SmHPPR1*, *SmPAL1*, *SmRAS1* and *SmTAT1*), transcription factors (*SmAREB1*, *SmbHLH148*, *SmbHLH37*, *SmbHLH51*, *SmERF115*, *SmERF1L1*, *SmMYB111*, *SmMYB36*, *SmMYB39*, *SmMYC2a*, *SmMYC2b*, *SmPAP1* and *SmTTG1*) and members of the SmMAPK family revealed two major clusters: one of these grouped *SmAREB1*, *SmERF115*, *SmMYB39*, *SmMYC2b*, *SmPAL1*, *SmPAP1* and *SmTTG1* with *SmMAPK2* and *SmMAPK5* through *SmMAPK10*, while the other comprised *SmbHLH148*, *SmbHLH51*, *SmbLH37*, *SmC4H1*, *SmCYP98A14 SmERF1L1*, *SmHPPR1*, *SmMYB111*, *SmMYB36*, *SmMYC2a*, *SmRAS1* and *SmTAT1* together with *SmMAPK3*, *SmMAPK4*, *SmMAPK13* and *SmMAPK15* through *SmMAPK18* (Fig. 5a). The former group formed two subclusters, one comprising *SmERF115*, *SmMYB39* and *SmPAP1* along with the SmMAPK members *SmMAPK1*, *SmMAPK2*, *SmMAPK6* through *SmMAPK9*, *SmMAPK11* and *SmMAPK12*; the other linked *SmAREB1*, *SmMYC2b*, *SmPAL1* and *SmTTG1* with *SmMAPK5* and *SmMAPK10* (Fig. 5a). The second major cluster formed four subclusters; in one of these subclusters, *SmMAPK3* was grouped with *SmbHLH51*, *SmC4H1*, *SmCYP98A14*, *SmERF11*, *SmHPPR1*, *SmMYB111*, *SmMYC2a*, *SmRAS1* and *SmTAT1*, while the other important subcluster grouped *SmbHLH37* and *SmbHLH148* with *SmMAPK4*, *SmMAPK13* and *SmMAPK15* through *SmMAPK17* (Fig. 5a). The similarity between the transcriptional behaviour of *SmbHLH51* and *SmMAPK3* implies that, given that *SmbHLH51* has been identified as a positive transcriptional regulator of phenolic acid synthesis [47], *SmMAPK3* very likely functions within the phenolic acid synthesis pathway (Fig. 5a).

The coexpression analysis directed toward genes encoding enzymes involved in the synthesis of tanshinones (*SmCPS1*, *SmCPS2*, *SmCYP76AH1*, *SmCYP76AH3*, *SmCYP76AK1*, *SmDXR*, *SmGGPPS1*, *SmHMGR1*, *SmHMGR2*, *SmIPI1* and *SmKSL1*), transcription factors (*SmAREB1*, *SmbHLH10*, *SmbHLH148*, *SmERF115*, *SmERF1L1, SmERF6*, *SmMYB36*, *SmMYB9*, *SmMYC2a*, *SmMYC2b* and *SmWRKY1*) and members of the SmMAPK family revealed a transcriptional relationship between *SmMAPK1*, *SmMAPK2*, *SmMAPK5* through *SmMAPK12*, *SmAREB1*, *SmCPS1*, *SmCPS2*, *SmCYP76AH1*, *SmCYP76AH3*, *SmCYP76AK1*, *SmDXR*, *SmERF5*, *SmGGPPS1*, *SmHMGR2*, *SmKSL1*, *SmMYC2* and *SmWRKY1* (Fig. 5b). Further linkage was noted between *SmMAPK3*, *SmMAPK4*, and *SmMAPK13* through *SmMAPK18* and *SmbHLH10*, *SmbHLH148*, *SmERF1L1*, *SmERF6*, *SmHMGR1*, *SmIPI1*, *SmMYB36*, *SmMYB9* and *SmMYC2a* (Fig. 5b). The former cluster resolved into three major subclusters. One of these comprised the genes encoding enzymes acting throughout the mevalonic acid and 2-C-methyl-D-erythritol-4-phosphate pathways (*SmCPS1*, *SmCYP76AH1*, *SmCYP76AH3*, *SmCYP76AK1*, *SmDXR*, *SmGGPPS1*, *SmHMGR2* and *SmKSL1*) along with *SmERF115* and the SmMAPK members *SmMAPK1*, *SmMAPK2*, *SmMAPK6* through *SmMAPK9*, *SmMAPK11* and *SmMAPK12*). The second cluster grouped genes encoding two upstream enzymes (*SmHMGR1* and *SmIPI1*) with those encoding seven transcription factors (*SmbHLH10*, *SmbHLH148*, *SmERF1L1*, *SmERF6*, S*mMYB36*, *SmMYB9* and *SmMYC2a*). Similar patterns of transcription were shown by the gene pairs *SmAREB1*/*SmMAPK5*, *SmWRKY1*/*SmMAPK10*, *SmERF115*/*SmMAPK2*, *SmHMGR1*/*SmMAPK16*, *SmbHLH148*/*SmMAPK13*, *SmbHLH10*/*SmMAPK14* and *SmMYB36*/*SmMAPK18* (Fig. 5b), implying the involvement of some *SmMAPK* products in the synthesis of tanshinones. The transcriptional behaviour of *SmIPI1*, *SmMYB9* and *SmMAPK3* was also quite similar.

As shown in Fig. 5, the expression of *SmMAPK14* (Group D) in different tissues/treatments was not significantly different. In addition, some gene expression preferences in different tissues/treatments could be observed from RNA-Seq data. For example, the expression of *SmMAPK4* (Group B) in different tissues (roots, flowers and leaves) and treatments (MeJA and YE) was more obvious than that under treatment with SA. Several group D members (*SmMAPK13*, *SmMAPK15* and *SmMAPK17*) showed higher gene expression levels in roots than in the treatments and other tissues, and *SmMAPK18* presented higher gene expression levels in flowers and leaves and under SA treatment. Interestingly, gene members in group A (*SmMAPK1*, *SmMAPK2* and *SmMAPK3*) presented higher expression levels than those in the other groups (Group B, Group C, and Group D). There are also mounting concerns that the expression of *SmMAPK3* in the roots and under treatment with MeJA, YE and SA is biased. In contrast, the expression of *SmMAPK1* in roots was lower than that under the treatments and in other tissues, and the expression of *SmMAPK2* was reduced after short-term treatments. This means that gene members in group A show complex responses to hormone treatments and complex regulatory mechanisms of phenolic acid and tanshinone synthesis under hormone treatments.

### Coexpression analysis of SmMAPKs and likely MAPK cascades acting in *S. miltiorrhiza*

**Fig. 6:**
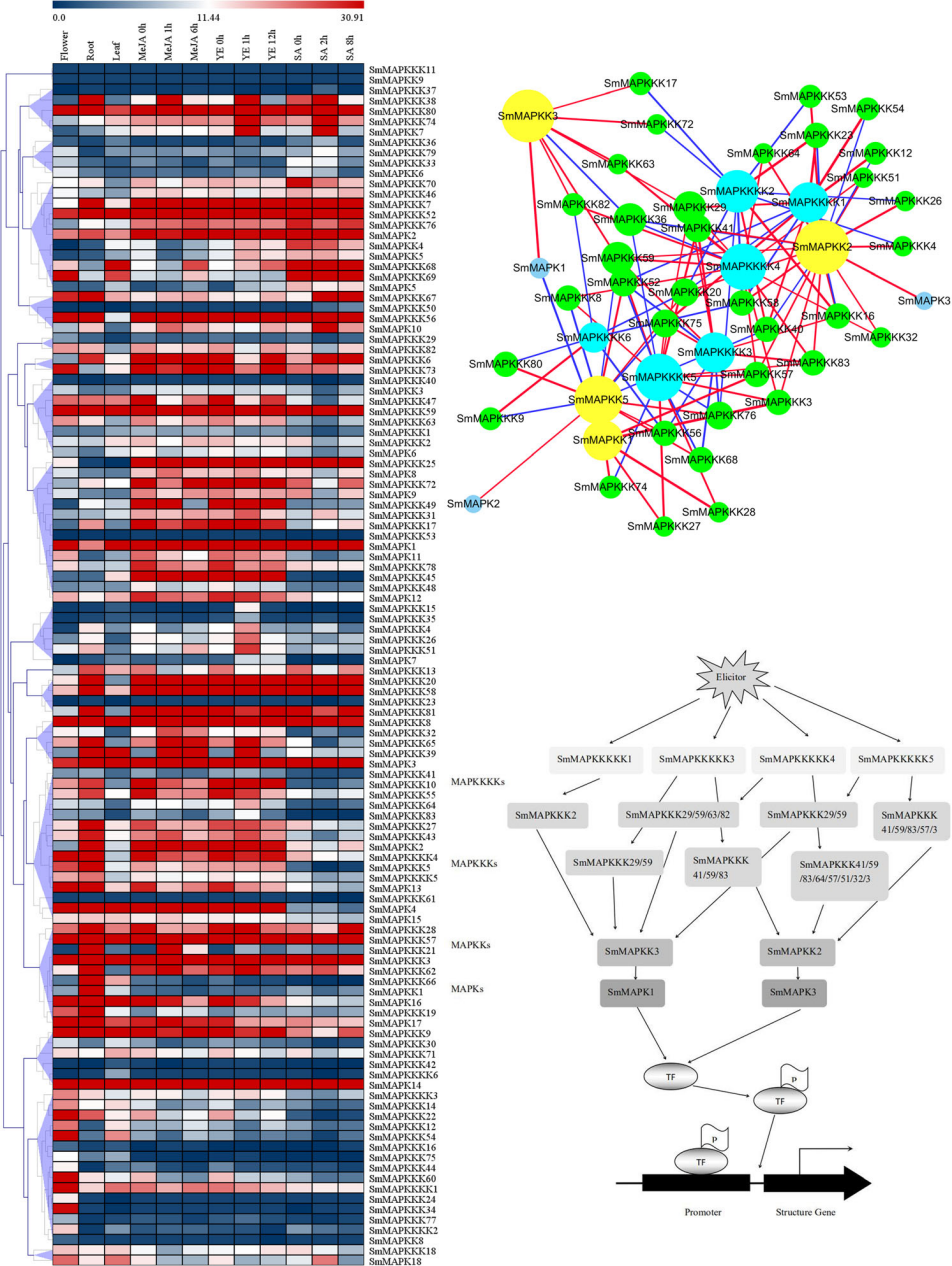
Probable MAPK cascades in *S. miltiorrhiza*. **a** Heat maps illustrating patterns of gene coexpression generated by Multi Experiment Viewer. Blue: low abundance, red: high abundance. **b** Network built on the basis of the correlation among SmMAPKKKKs, SmMAPKKKs, SmMAPKKs and SmMAPKs. Pearson correlation coefficient (PCC) values were calculated for each pair of genes. MAPK cascade reaction map constructed using Cytoscape 3.6.1.0 software. **c** MAPK cascade pattern diagram

The results of the coexpression analysis of the *SmMAPKs* family are shown in Fig. 6a, and the interaction network of the MAPK cascades is presented in Additional file 2 Figure S2. Based on the situation in *A. thaliana*, the expectation was that there would also be two MAPK cascades in *S. miltiorrhiza*, one related to *AtMPK6* and the other to *AtMPK3*. However, the analysis implied that there were three, related to *SmMAPK1* (84.8% similar to *AtMPK6*), *SmMAPK2* (likely homologue of *AtMPK6*) and *SmMAPK3* (likely homologue of *AtMPK3*). The interaction network shown in Fig. 6b involved two cascades: the participants in the first were *SmMAPK1*, *SmMAPKK3*, four *SmMAPKKKs* (*SmMAPKKK29*, *SmMAPKKK59*, *SmMAPKKK63*, *SmMAPKKK82*) and four *SmMAPKKKKs* (*SmMAPKKKK1*, *SmMAPKKKK3* through *SmMAPKKKK5*), and those in the second were *SmMAPK3*, *SmMAPKK2*, eight *SmMAPKKKs* (*SmMAPKKK3*, *SmMAPKKK32*, *SmMAPKKK41*, *SmMAPKKK51*, *SmMAPKKK57*, *SmMAPKKK59*, *SmMAPKKK64* and *SmMAPKKK83*) and three *SmMAPKKKKs* (*SmMAPKKKK3* through *SmMAPKKKK5*) (Fig. 6c). SmMAPK1 might be activated by SmMAPKK3, which is in turn probably activated by SmMAPKKK29, SmMAPKKK59, SmMAPKKK63 and SmMAPKKK82; these MAPKKKs could be activated by MAPKKK kinases (SmMAPKKKK1 and SmMAPKKKK3 through SmMAPKKKK5). On the other hand, SmMAPKK2 might be activated by the MAPKK kinases SmMAPKKK3, SmMAPKKK32, SmMAPKKK41, SmMAPKKK51, SmMAPKKK57, SmMAPKKK59, SmMAPKKK64 and SmMAPKKK83, which are in turn probably activated by the MAPKKK kinases SmMAPKKKK1 and SmMAPKKKK3 through SmMAPKKKK5. These proteins phosphorylate SmMAPKK2, which in turn phosphorylates SmMAPK3 (Fig. 6c).

**Fig. 7:**
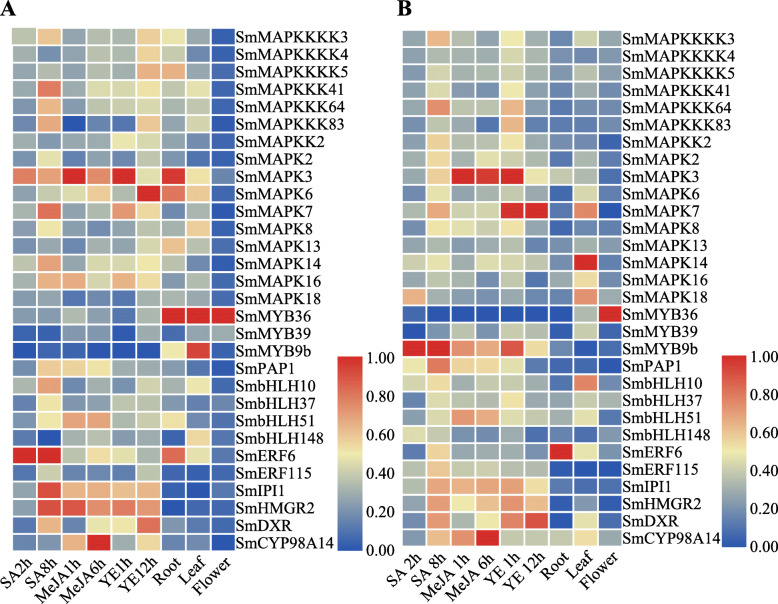
qRT-PCR analyses of coexpressed genes. **a** Heat maps of coexpressed genes based on qRT-PCR analyses. **b** Heat maps of coexpressed genes based on RNA-Seq analyses

To validate the results of the coexpression analysis, we used quantitative RT-PCR. A total of 30 genes were tested, including MAPK cascade genes, enzymes and transcription factors (TFs). The correlation coefficient of the R value between the Ct value of the qRT-PCR results and the log_2_^RPKM^ values from the RNA-seq analysis was calculated for each gene via Pearson correlation. The results are presented in Additional file 1 Figure S3. It was clear that the expression of *SmMAPK3* according to qRT-PCR was similar to that according to RNA-Seq (Fig. 7). Furthermore, TFs (*SmERF6*/*SmERF115*) and enzymes (*SmIPI1*/*SmHMGR2*/*SmDXR/SmCYP98A14*) showed the same pattern (Fig. 7). In addition, Fig. 7 shows that the pattern differed between the qRT-PCR and RNA-seq data for some genes, such as *SmMAPK3* in roots, *SmMYB36* in roots and leaves and *SmMAPK6* under YE treatment. The minor difference between the qRT-PCR and RNA-seq might be caused by two experimental systems. It was normal also because the site where we harvested the plant material differed from the site where the materials used to generat the RNA-seq data were collected. This is an inevitable error because we could not obtain the same samples used in the other analyses.

### SmMAPK3 directly interacts with SmMYBs and SmAREB1

**Fig. 8:**
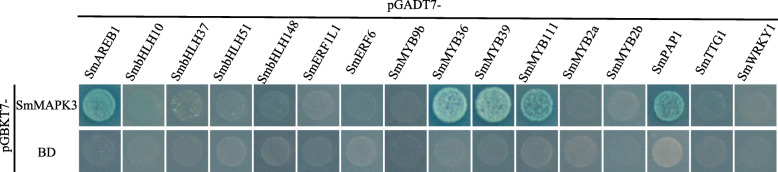
SmMAPK3 physically interacts with SmMYBs and SmAREB1. Y2H assay to detect the interactions of SmMAPK3 with SmAREB1, SmbHLH10, SmbHLH37, SmbHLH51, SmbHLH148, SmERF1L1, SmERF6, SmMYB9b, SmMYB36, SmMYB39, SmMYB111, SmMYC2a, SmMYC2b, SmPAP1, SmTTG1 and SmWRKY1. Transformed yeast was grown on selective medium lacking adenine, histidine, leucine, and tryptophan (SD-LWHA) with AbA and x-a-gal to test protein interactions

For the Y2H assay, *SmMAPK3* was fused to pGBKT7, and *SmMYC2a*, *SmMYC2b*, *SmMYB36*, *SmMYB39*, *SmMYB111*, *SmMYB9b*, *SmPAP1*, *SmTTG1*, *SmWRKY1*, *SmAREB1*, *SmERF6*, *SmHLH51*, *SmHLH10*, *SmHLH148*, *SmHLH37* and *SmERF1L1* were ligated to pGADT7 to generate pGBKT7-SmMAPK3 and pGADT7-TF. The Y2H Gold strain yeast cells transformed with BD-SmMAPK3 and AD-SmMYC2a/SmMYC2b/SmMYB9b/SmTTG1/SmWRKY1/SmERF6/SmHLH51/SmHLH10/SmHLH148/SmHLH37/SmERF1L1 could not grow on SD-LWHA with AbA and X-ɑ-Gal, while the Y2H Gold strain yeast cells cotransformed with BD-SmMAPK3 and AD-SmMYB36/SmMYB39/SmMYB111/SmPAP1/SmAREB1 could grow on SD-LWHA with AbA and expressed Mel1 and turned blue in the presence of the X-α -Gal substrate (Fig. 8).

## Discussion

A number of studies have focused on systematically identifying and characterizing the proteins participating in plant MAPK cascades [48–51], which affect various aspects of both growth and development [18, 52, 53], stress tolerance [23, 24, 34, 54] and the synthesis of secondary metabolites [17, 26, 33]. The present research has extended the scope of these studies to the medicinal species *S. miltiorrhiza*, made possible by the recent acquisition of its genomic sequence [46]. Our signal outcome was the identification of six *SmMAPKKKKs*, 83 *SmMAPKKKs*, nine *SmMAPKKs*, and 18 *SmMAPK* genes containing conserved domains through genome-wide analysis. At the same time, cascades involving the participation of *SmMAPK3* and *SmMAPK1* were identified, which were most likely to be involved in phenolic acid and tanshinone biosynthesis according to an enzyme and TF gene-to-MAPK cascade gene correlation analysis.

Through the exploitation of RNA-seq-based data, it was possible to correlate the transcriptional profiles of genes encoding both key enzymes and transcription factors with members of the *SmMAPK* gene family. For example, the profiles of the two enzyme-encoding genes *SmHMGR1* and *SmIPI1* resembled those of seven transcription factor-encoding genes (*SmbHLH10*, *SmbHLH148*, *SmERF1L1*, *SmERF6*, S*mMYB36*, *SmMYB9* and *SmMYC2a*) (Fig. 5b). Note that SmIPI1 has been identified as a key enzyme in the terpenoid metabolic pathway [55]; the correlation of the transcription of *SmMAPK3* with that of *SmIPI1* suggested that the product of the former gene may be involved in the regulation of tanshinone synthesis. The product of SmERF6 modulates the synthesis of tanshinones through its binding to a GCC box present in the promoters of both *SmCPS1* and *SmKSL1* [56], while SmMYB36 interacts with many MYB-related core elements (MBSI, MBSII, AAAAAAC(C/G) GTTA, CAACTG and AAAAGTTAGTTA) present in the promoters of various genes encoding enzymes involved in the synthesis of secondary metabolites [57]. Correlations were also identified for a set of eight enzyme-encoding genes (*SmCPS1*, *SmCYP76AH1*, *SmCYP76AH3*, *SmCYP76AK1*, *SmDXR*, *SmGGPPS1*, *SmHMGR2* and *SmKSL1*) along with three genes encoding transcription factors (*SmAREB1*, *SmERF115* and *SmWRKY1*). SmWRKY1 has been shown to participate in the regulation of tanshinone synthesis through its interaction with SmDXR [58]. Another set of correlated genes included six genes encoding transcription factors (*SmAREB1*, *SmERF115*, *SmMYB3*, *SmMYC2b*, *SmPAP1* and *SmTTG1*) and one gene encoding an enzyme (*SmPAL1*) (Fig. 5a). SmAREB1 has been demonstrated to promote the flux of metabolites through the phenolic acid-branched pathway via its phosphorylation of SmSnRK2.6 [59]. The overexpression of *SmMYC2* promotes the production of Sal B [60]. AtMPK6, which is phosphorylated by AtMKK3 (MAPK kinase 3), phosphorylates a basic helix-loop-helix transcription factor, AtMYC2. Furthermore, AtMYC2 binds to the AtMPK6 promoter and regulates its expression in a feedback regulatory mechanism in blue light signalling [61]. Therefore, SmMAPK cascades probably phosphorylate SmMYC2 or other TFs, and the TFs then regulate the expression of enzyme genes. *SmMAPK2*, *SmMAPK3*, *SmMAPK5*, *SmMAPK8*, *SmMAPK10*, *SmMAPK13* and *SmMAPK18* shared a similar transcriptional profile with a number of genes encoding either enzymes or transcription factors associated with the synthesis of phenolic acids, as did *SmMAPK2*, *SmMAPK3*, *SmMAPK5*, *SmMAPK10*, *SmMAPK13*, *SmMAPK14*, *SmMAPK16* and *SmMAPK18* with genes encoding either enzymes or transcription factors associated with the synthesis of tanshinones (Fig. 5) Nine of the 18 SmMAPK proteins (SmMAPK2, SmMAPK3, SmMAPK5, SmMAPK8, SmMAPK10, SmMAPK13, SmMAPK14, SmMAPK16 and SmMAPK18) are potentially involved in the synthesis of key secondary metabolites; six of these proteins (SmMAPK2, SmMAPK3, SmMAPK5, SmMAPK10, SmMAPK13 and SmMAPK18) potentially participate in the synthesis of both phenolic acids and tanshinones, while SmMAPK8 is involved in the synthesis of only phenolic acids, and SmMAPK14 and SmMAPK16 are involved in the synthesis of nonphenolic acids but not phenolic acids. It is proposed that SmMAPK2, SmMAPK3, SmMAPK5, SmMAPK8 and SmMAPK10 are positive regulators, while SmMAPK18 is a negative regulator of phenolic acid synthesis; SmMAPK3, SmMAPK5, SmMAPK10, SmMAPK13, SmMAPK14, SmMAPK16 and SmMAPK18 also act to promote tanshinone synthesis.

Elicitors such as yeast elicitors, metal ions (Ag^+^, Cu^2+^, Zn^2+^, Co^2+^), plant growth regulators (SA, ABA, ETH, MeJA, IAA, NAA, GA, 6-BA, TDZ), and other treatments (polyamines, ultraviolet-B radiation, H_2_O_2_), induce the biosynthesis and accumulation of secondary metabolites (tanshinone and phenolic acid biosynthesis) in *S. miltiorrhiza* [11, 13], especially plant hormones (MeJA, SA, ETH, ABA, GA) [62–64]. MAPK cascades have also been implicated in ABA, ETH, JA and SA signalling [17, 25, 29, 33, 36, 37]. ABA, ETH, JA and SA are important plant hormones, and their crosstalk is crucial for secondary metabolite biosynthesis during defence against pathogens and insects [65, 66]. The present analysis suggested that in *S. miltiorrhiza*, members of group A (*SmMAPK1*, *SmMAPK2* and *SmMAPK3*) show a higher expression level under treatments with MeJA, YE and SA than the other groups (Group B, Group C, and Group D) (Fig. 5), and SmMAPK1 and SmMAPK3 are both probably important regulators of secondary metabolite synthesis (Fig. 6c). SmMAPK3 can physically interact with SmMYB36/SmMYB39/SmMYB111/SmPAP1/SmAREB1 (Fig. 8), which have been reported to regulate the synthesis and accumulation of secondary metabolites [57, 59, 67–69]. In *A. thaliana*, MAPKK9 promotes ethylene and camalexin biosynthesis [70], and both AtMPK3 and AtMPK6, which are highly involved in the plant response to biotic [71–75] and abiotic stress [21, 54, 76] and the regulation of ETH [17, 26, 33], SA [29, 75] and JA [33, 77] production, are activated by the upstream regulatory MAPK kinases AtMKK4 and AtMKK5 [78, 79], which are in turn regulated by the upstream MAPKK kinase AtMEKK1 [80]. Such interspecific similarity in MAPK function and interspecific differences in the architecture of MAPK cascades explain the conservation and variability of gene evolution.

To further identify and characterize the functions of candidate genes (such as *SmMAPK3*), we plan to overexpress and conducted RNA interference knockdown of *SmMAPK3* to obtain an initial understanding of its function in phenolic acid and tanshinone synthesis. Thereafter, we will validate the proteins interacting with *SmMAPK3* (either screened in Y2H assays or reported in the literature). Then, we will verify the function of the interacting proteins and determine their upstream and downstream relationships. Ultimately, we will be able to improve the quality of *S. miltiorrhiza* through genetic modification.

## Conclusions

The present study has provided an exhaustive catalogue of the genes encoding MAPKs, MAPKKs, MAPKKKs and MAPKKKKs in *S. miltiorrhiza*: 18 genes were identified as putatively encoding MAPKs, nine MAPKKs, 83 MAPKKKs and six MAPKKKKs. The set of *SmMAPKKKKs* formed two subfamilies (GCK-III, GCK-VI), the *SmMAPKKKs* three subfamilies (MEKK, ZIK and RAF), the *SmMAPKKs* five subfamilies (A through E) and the *SmMAPKs* four subfamilies (groups A through D). The identity of each subfamily was supported by its sequence-based phylogeny, by the exon-intron structure of its member genes and by the content of conserved domains/motifs. Most of these genes were transcriptionally active in the roots, leaves and flowers of the *S. miltiorrhiza* plant, although there was some evidence of topological specialization of a few of these genes. The results of quantitative RT-PCR verified that the gene coexpression analysis based on the RNA-seq data was accurate. Group A of the *SmMAPK* genes appeared to be inducible, not only by elicitors such as yeast extract but also by phytohormones such as salicylic acid and jasmonate. They appear to be prominently expressed during the defence response and the synthesis of secondary metabolites. According to the Y2H assay, SmMAPK3 physically binds TFs including SmMYB36, SmMYB39, SmMYB111, SmPAP1 and SmAREB1, probably to regulate the synthesis of secondary metabolites.

## Methods

### Plant materials

Three different *S. miltiorrhiza* tissues, the roots, flowers, and leaves, were collected from the botanical garden of *S. miltiorrhiza* at Northwest A&F University in Yangling, China. *S. miltiorrhiza* hairy roots (0.3 g FW) resulting from the infection of sterile plantlets with *Agrobacterium rhizogenes* (*ATCC15834*) (WEIDI, Shanghai, China) were cultured in a 100 ml shake flask containing 50 ml of liquid 6,7-V medium on an orbital shaker.

### Gene discovery and bioinformatic analysis

Hidden Markov model profiles were built using HMMER v.3.1 software [81] (www.hmmer.org) with the aim of identifying MAPK cascade family sequences in the *S. miltiorrhiza* genome. The query sequences from *A. thaliana* (the sequences and Accession Numbers are in Additional file 1 Table S6) comprised 20 genes encoding MAPKs, 10 MAPKKs, 80 MAPKKKs and 10 MAPKKKKs [15, 16]. The applied E value threshold was 10^− 6^. After the manual removal of redundant sequences, alignment was performed using the Clustal W program [82]. A phylogenetic analysis was conducted using MEGA v7.0 software [83] (www.megasoftware.net) applying the neighbour-joining method [84] and a 500 replication bootstrap test. The molecular weight and pI of each gene product were predicted using the Compute pI/MW tool mounted on the ExPASy server [85] (www.expasy.org). The members of MAPK cascades were also subjected to analysis based on MEME software [86] (meme-suite.org/tools/meme). Gene structures were obtained using Gene Structure Display Server 2.0 (gsds.cbi.pku.edu.cn/). Conserved domains were identified using DNAMAN software (https://www.lynnon.com/). Sequences upstream of the transcription start site (ATG) of the SMMAPKs were derived from the *S. miltiorrhiza* genome sequence. The cis-acting element content of these promoter-containing sequences was deduced using the PLACE database (www.dna.affrc.go.jp/PLACE/signalscan.html).

### Coexpression analyses

RNA-seq reads were derived from mRNA extracted from the leaves, flowers and roots of plants subjected to three treatments (salicylic acid, methyl jasmonate and yeast extract). The reads were recovered from the Sequence Read Archive (SRA) (www.ncbi.nlm.nih.gov/sra) under accession numbers SRR1043998, SRR1045051, SRR1020591, SRX1423774, SRX2992229, SRX2992230, SRX2992231, SRX2992232 and SRX2992233. RPKMs, calculated using the BMKCloud tool (www.biocloud.net), were used to derive the Pearson correlation coefficient for each pair of transcripts using the bivariate correlation analysis tool implemented in Excel2010; correlations > 0.5 were considered significant.

Coexpression/coregulation cluster analysis was performed for samples from 12 different tissues or time point by MeV (Version 4.9) [87]. The normalized expression values of the genes were calculated by dividing their expression levels from different tissues or time points. Hierarchical clustering (HCL) was performed using MeV with default settings. The MAPK cascade reaction map was constructed with Cytoscape 3.6.1.0 software (https://cytoscape.org/).

### RNA extraction and gene expression analysis

For the quantitative real-time PCR (qRT-PCR) analysis of MAPK cascade genes and their coexpressed genes in *S. miltiorrhiza*, total RNA was extracted from the leaves, flowers, and roots of plants and Danshen hairy roots treated for different times with salicylic acid, methyl jasmonate and yeast extract [88]. Total RNA was isolated by using the RNAprep Pure Plant Kit (TIANGEN, Beijing, China) according to the manufacturer’s instructions. cDNA was prepared from total RNA by using the PrimeScript RT reagent kit (TaKaRa, Dalian, Chain). For every sample, qRT-PCR was performed on a real-time PCR system (Bio-RAD CFX96, CA, USA) with the TB Green® Premix Ex TaqTM II Kit (TaKaRa, China). Gene-specific primers (Additional file 1 Table S7) were designed with Primer Premier v5.0 software to detect the expression of relevant genes. The expression levels of target genes were normalized to those of β-actin and ubiquitin [59].

### Yeast two-hybrid (Y2H) assays

The coding sequence of the *SmMAPK3* gene was cloned into the pGBKT7 vector, and those of *SmMYC2a*, *SmMYC2b*, *SmMYB36*, *SmMYB39*, *SmMYB111*, *SmMYB9b*, *SmPAP1*, *SmTTG1*, *SmWRKY1*, *SmAREB1*, *SmERF6*, *SmHLH51*, *SmHLH10*, *SmHLH148*, *SmHLH37* and *SmERF1L1* were cloned into pGADT7. The plasmids were transformed into Y2H Gold yeast cells and grown on SD-dropout medium lacking leucine and tryptophan (SD-LW) medium. Furthermore, yeast cells were screened on SD-selection medium lacking adenine, histidine, leucine, and tryptophan (SD-LWHA) with aureobasidin A (AbA) and a-galactosidase (X-ɑ-Gal). Interactions were observed after a 3 d of incubation at 30 °C.

### Statistical analysis

All statistical calculations were performed using routines implemented in SPSS v18.0 software; the chosen significance thresholds were *P* < 0.05 and < 0.01.

## Supplementary information

**Additional file 1 :Table S1.** Sequences belonging to the SmMAPK family. **Table S2**. Summary data of the set of genes belonging to the SmMAPK family. **Table S3.** The content of cis-acting elements present in the promoter sequences associated with genes belonging to the SmMAPK family. **Table S4.** Enzymes participating in the synthesis of phenolic acids and tanshinones in *S. miltiorrhiza*. **Table S5.** Transcription factors regulating the synthesis of phenolic acids and tanshinones in *S. miltiorrhiza*. **Table S6.** The query sequences from *A. thaliana*. **Table S7.** Primers used for qRT-PCR and vector construction.

**Additional file 2 :Figure S1.** Conserved motifs in the products of genes belonging to the SmMAPK family, as predicted by MEME software. **Figure S2.** A proposed interaction network involving the products of genes belonging to the SmMAPK family. **Figure S3.** Pearson correlation coefficient of R value between the Ct value of the qRT-PCR results and the log2 RPKM values from the RNA-seq analysis. Pearson correlation coefficient of R values were visualized by TBtools.

## Data Availability

The DNA sequencing data and the Protein sequences are available in the National Data Center of Traditional Chinese Medicine of China database with the link of ftp://danshen.ndctcm.org:10402/, under the gene ID provided in Additional file 1 Table S1. The query sequences from *A. thaliana* are available in the National Center for Biotechnology Information (NCBI) database with the link of https://www.ncbi.nlm.nih.gov/, under the accession number provided in Additional file 1 Table S6. And the RNA sequencing data are available in the Sequence Read Archive (SRA) database with the link of www.ncbi.nlm.nih.gov/sra, under the accession number SRR1043998, SRR1045051, SRR1020591, SRX1423774, SRX2992229, SRX2992230, SRX2992231, SRX2992232 and SRX2992233, respectively. All of the datasets supporting the results of this article are included within the article and its Additional files.

## Abbreviations

ABA: Abscisic acid
C4H: Cinnamate 4-hydroxylase
HPPR: 4-Hydroxyphenylpyruvate reductase
JA: Jasmonic acid
MAPK cascades: Mitogen-activated protein kinase cascades
MAPK: Mitogen-activated protein kinase
MAPKK: MAPK kinase
MAPKKK: MAPK kinase kinase
MAPKKKK: MAPK kinase kinase kinase kinase
MeJA: Methyl jasmonate
MEME: Multiple expectation maximization for motif elicitation
NCBI: National Center for Biotechnology Information
PAL: Phenylalanine ammonia-lyase
RAS: Rosmarinic acid synthase
SA: Salicylic acid
TAIR: The Arabidopsis information resource
TAT: Tyrosine aminotransferase
4CL: 4-Coumaric acid CoA-ligase
