# Supplementary material for: Genome-wide characterization and expression profiling of MAPK cascade genes in Salvia miltiorrhiza reveals the function of SmMAPK3 and SmMAPK1 in secondary metabolism
Source: BMC Genomics. 2020 Sep 14;21:630. doi: 10.1186/s12864-020-07023-w (PMC7488990; doi:10.1186/s12864-020-07023-w)
Supplement: Supplementary file 2 — Additional file 2 :Figure S1. Conserved motifs in the products of genes belonging to the SmMAPK family, as predicted by MEME software. Figure S2. A proposed interaction network involving the products of genes belonging to the SmMAPK family. Figure S3. Pearson correlation coefficient of R value between the Ct value of the qRT-PCR results and the log2 RPKM values from the RNA-seq analysis. Pearson correlation coefficient of R values were visualized by TBtools. [file 12864_2020_7023_MOESM2_ESM.pdf]

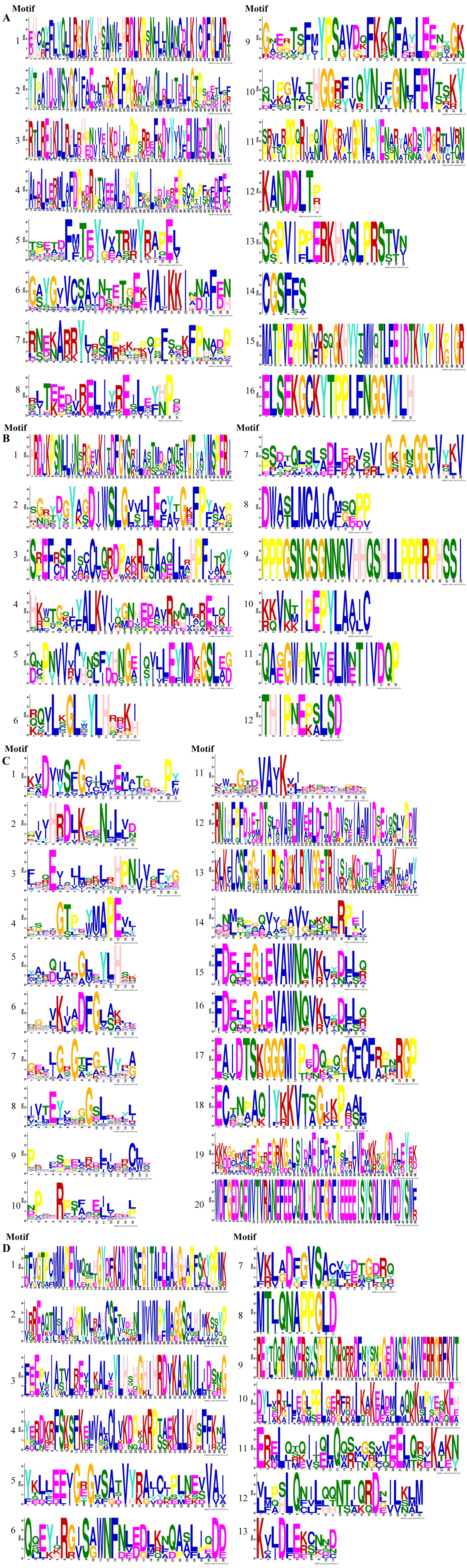

Figure S1: Conserved motifs in the products of genes belonging to the SmMAPK family, as predicted by MEME software. (A) The conserved motifs of SmMAPK

detected by the online tool MEME, (B) The conserved motifs of SmMAPKK detected by the online tool MEME, (C) The conserved motifs of SmMAPKKK detected by the online tool MEME, (D) The conserved motifs of SmMAPKKKK detected by the online tool MEME.

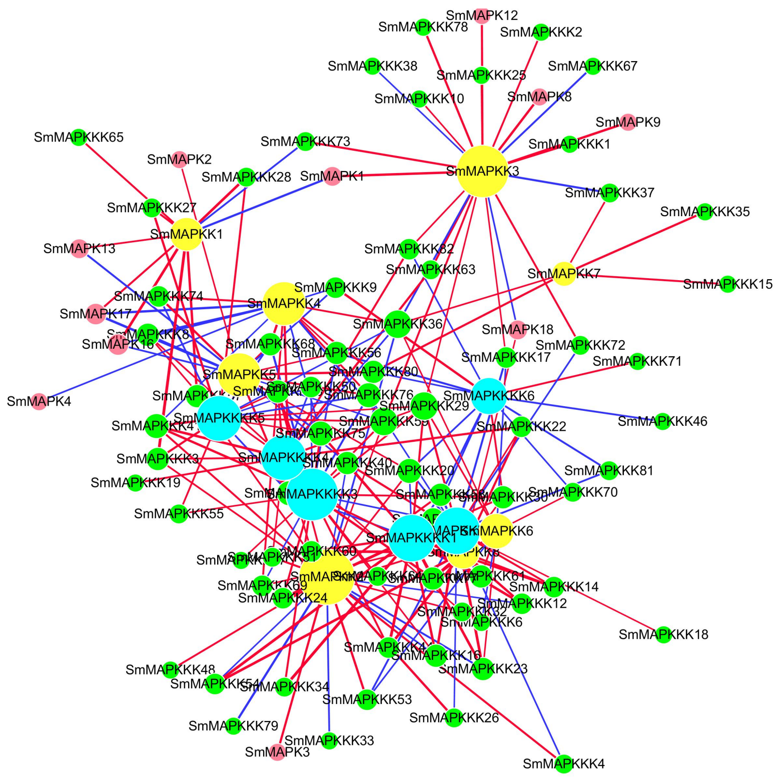

Figure S2. A proposed interaction network involving the products of genes belonging to the SmMAPK family. MAPK cascades reaction map was constructed with cytoscape 3.6.1.0 using Pearson correlation coefficient

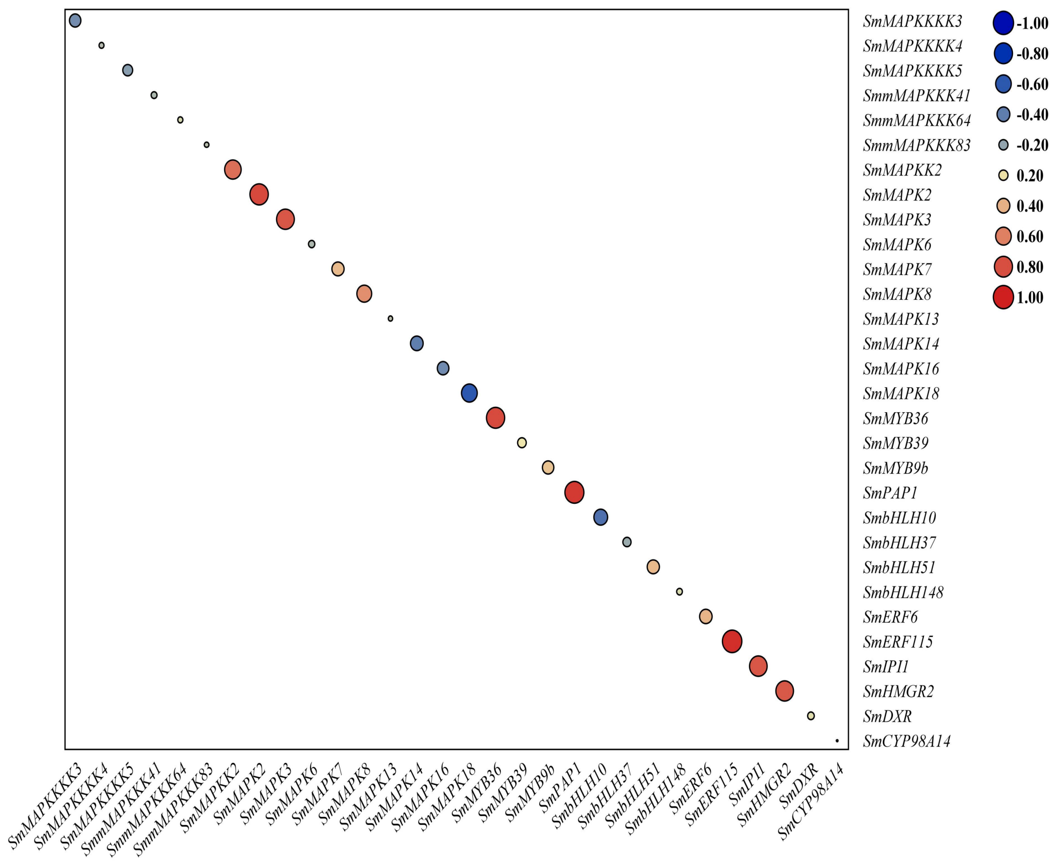

Figure S3: Pearson correlation coefficient of R value between the Ct value of the qRT-PCR results and the $\log_2$ ^{RPKM} values from the RNA-seq analysis. Pearson correlation coefficient of R values were visualized by TBtools.
